# Accuracy of Mobile Phone and Handheld Light Microscopy for the Diagnosis of Schistosomiasis and Intestinal Protozoa Infections in Côte d’Ivoire

**DOI:** 10.1371/journal.pntd.0004768

**Published:** 2016-06-27

**Authors:** Jean T. Coulibaly, Mamadou Ouattara, Michael V. D’Ambrosio, Daniel A. Fletcher, Jennifer Keiser, Jürg Utzinger, Eliézer K. N’Goran, Jason R. Andrews, Isaac I. Bogoch

**Affiliations:** 1 Unité de Formation et de Recherche Biosciences, Université Félix Houphouët-Boigny, Abidjan, Côte d’Ivoire; 2 Centre Suisse de Recherches Scientifiques en Côte d’Ivoire, Abidjan, Côte d’Ivoire; 3 Swiss Tropical and Public Health Institute, Basel, Switzerland; 4 University of Basel, Basel, Switzerland; 5 Department of Bioengineering, University of California–Berkeley, Berkeley, California, United States of America; 6 Division of Infectious Diseases and Geographic Medicine, Stanford University School of Medicine, Stanford, California, United States of America; 7 Divisions of Internal Medicine and Infectious Diseases, Toronto General Hospital, Toronto, Canada; 8 Department of Medicine, University of Toronto, Toronto, Canada; George Washington University, UNITED STATES

## Abstract

**Background:**

Handheld light microscopy using compact optics and mobile phones may improve the quality of health care in resource-constrained settings by enabling access to prompt and accurate diagnosis.

**Methodology:**

Laboratory technicians were trained to operate two handheld diagnostic devices (Newton Nm1 microscope and a clip-on version of the mobile phone-based CellScope). The accuracy of these devices was compared to conventional light microscopy for the diagnosis of *Schistosoma haematobium*, *S*. *mansoni*, and intestinal protozoa infection in a community-based survey in rural Côte d’Ivoire. One slide of 10 ml filtered urine and a single Kato-Katz thick smear from 226 individuals were subjected to the Newton Nm1 microscope and CellScope for detection of *Schistosoma* eggs and compared to conventional microscopy. Additionally, 121 sodium acetate-acetic acid-formalin (SAF)-fixed stool samples were examined by the Newton Nm1 microscope and compared to conventional microscopy for the diagnosis of intestinal protozoa.

**Principal Findings:**

The prevalence of *S*. *haematobium*, *S*. *mansoni*, *Giardia intestinalis*, and *Entamoeba histolytica/E*. *dispar*, as determined by conventional microscopy, was 39.8%, 5.3%, 20.7%, and 4.9%, respectively. The Newton Nm1 microscope had diagnostic sensitivities for *S*. *mansoni* and *S*. *haematobium* infection of 91.7% (95% confidence interval (CI) 59.8–99.6%) and 81.1% (95% CI 71.2–88.3%), respectively, and specificities of 99.5% (95% CI 97.0–100%) and 97.1% (95% CI 92.2–99.1%), respectively. The CellScope demonstrated sensitivities for *S*. *mansoni* and *S*. *haematobium* of 50.0% (95% CI 25.4–74.6%) and 35.6% (95% CI 25.9–46.4%), respectively, and specificities of 99.5% (95% CI 97.0–100%) and 100% (95% CI 86.7–100%), respectively. For *G*. *intestinalis* and *E*. *histolytica/E*. *dispar*, the Newton Nm1 microscope had sensitivity of 84.0% (95% CI 63.1–94.7%) and 83.3% (95% CI 36.5–99.1%), respectively, and 100% specificity.

**Conclusions/Significance:**

Handheld diagnostic devices can be employed in community-based surveys in resource-constrained settings after minimal training of laboratory technicians to diagnose intestinal parasites.

## Author Summary

Handheld light microscopes are new technologies that may be helpful in enabling better access to diagnostic testing for people living in resource-constrained settings in tropical and subtropical countries. Recent studies evaluating the accuracy of such devices have focused on their use by expert microscopists and were mainly conducted in laboratories. We evaluated the operating performance of two handheld microscopes (Newton Nm1 microscope and clip-on version of the reversed-lens CellScope) in comparison to conventional microscopy for the diagnosis of urogenital and intestinal schistosomiasis, when integrated into routine use in a community-based survey carried out in Côte d’Ivoire. Additionally, we evaluated the same microscopist’s diagnostic performance with the Newton Nm1 microscope for intestinal protozoa in a laboratory set-up. The Newton Nm1 microscope demonstrated excellent diagnostic sensitivity and specificity for schistosomiasis and intestinal protozoa. The CellScope had high specificity but only modest sensitivity for schistosomiasis diagnosis. Taken together, handheld diagnostic tools show promise to improve the quality of clinical and public health care delivered in resource-constrained settings.

## Introduction

Neglected tropical diseases have considerable detrimental impacts in resource-constrained settings as they can result in chronic disability and stigmatization, and have profound negative economic consequences [1,2]. Microscopy is an essential tool in the diagnosis and surveillance of many neglected tropical diseases and is a vital component in virtually every clinical and public health laboratory worldwide. Unfortunately even basic microscopy facilities are lacking in many resource-constrained settings where the greatest needs exist, and where neglected tropical diseases are rife [3].

Schistosomiasis is a neglected tropical disease and an important public health threat, with countries in sub-Saharan Africa affected most [4]. Chronic infection with *Schistosoma mansoni* may result in disability and death due to complications of portal hypertension, while chronic infection with *S*. *haematobium* frequently results in genitourinary morbidity and mortality, with bladder cancer as a well-known complication [5,6]. Intestinal protozoa, such as *Entamoeba histolytica* and *Giardia intestinalis*, are common pathogens accounting for widespread morbidity and mortality in resource-constrained settings. For example, *E*. *histolytica* is responsible for an estimated 40,000 to 100,000 deaths annually. *G*. *intestinalis*, a common cause of diarrheal illness, has an estimated prevalence of 20–30% in low-income countries [7,8]. These infections are diagnosed primarily by stool microscopy (or urine microscopy in the case of *S*. *haematobium*).

Recently, portable handheld microscopes [9–11] and mobile phone-based microscopes [12–14] have been evaluated for the diagnosis of neglected tropical diseases (e.g., schistosomiasis, opisthorchiasis, and soil-transmitted helminthiasis) and malaria. Most of the prior studies evaluating handheld and mobile phone-based microscopy have utilized expert microscopists in field settings, or were implemented under laboratory conditions. Prior to wide-scale utilization, these devices must be validated in real-world clinical and public health settings, and operated by individuals who will use them in routine daily practice. Here, we integrate a handheld light microscope (i.e., Newton Nm1 microscope; Newton Microscopes; Cambridge, United Kingdom) [15] and a handheld mobile phone-based microscope (i.e., clip-on version of the reversed-lens CellScope) [16] for the diagnosis of *S*. *mansoni*, *S*. *haematobium*, and intestinal protozoa into a community-based survey in rural Côte d’Ivoire. These devices were chosen because of their compact design, ease of use, and sufficient resolution to detect intestinal and urogenital parasites. We assessed the accuracy of these devices by comparing them to routine microscopy.

## Methods

### Ethics Statement

This study was embedded into a larger, cross-sectional, community-based survey in Côte d’Ivoire. Ethical approval was granted by the Ministry of Health and Public Hygiene of Côte d’Ivoire (reference no., 32/MSLS/CNER-dkn). Written informed consent was obtained from adults aged 18 years or older, and parents or legal guardians on behalf of children. Children, in addition, assented orally. Anthelmintic treatment was offered to all participants at the end of the study (i.e., praziquantel, 40 mg/kg of body weight for schistosomiasis and albendazole, 400 mg for soil-transmitted helminthiasis).

### Study Design and Population

This cross-sectional study was conducted in the village of Grand Moutcho in southern Côte d’Ivoire (geographic coordinates: 4.181 N latitude and 5.961 E longitude). The village belongs to a region that is highly endemic for schistosomiasis [17]. The study was carried out between April and June 2014. Study participants were between 6 and 19 years of age.

### Field Procedures

Early morning stool and urine samples, collected between 10:00 and 12:00 hours [18], were processed and evaluated on the spot in a community clinic. Fresh stool samples were processed with the Kato-Katz technique [19]. In brief, standard 41.7 mg thick smears were placed on microscope slides for evaluation of *S*. *mansoni* and soil-transmitted helminth eggs. In addition, approximately 2 g of unprocessed stool from each individual was fixed in a standard solution of sodium acetate-acetic acid-formalin (SAF) for subsequent laboratory processing and diagnosis of intestinal protozoa infections. Urine samples were first shaken, then 10 ml was extracted and pressed through a 13 mm diameter meshed filter with 20 μm pores (Sefar AG; Heiden, Switzerland). One drop of Lugol’s iodine solution was placed over the filter prior to examination.

### Microscopy Evaluation of *Schistosoma* and Soil-Transmitted Helminths

We selected one Kato-Katz thick smear slide and one filtered urine slide from each individual on their first day of participation in the study. Each slide was subjected to three microscope techniques shortly after collection, and eggs were identified and quantified at the field site. All microscopists were blinded to prior diagnoses on each slide.

**Fig 1:**
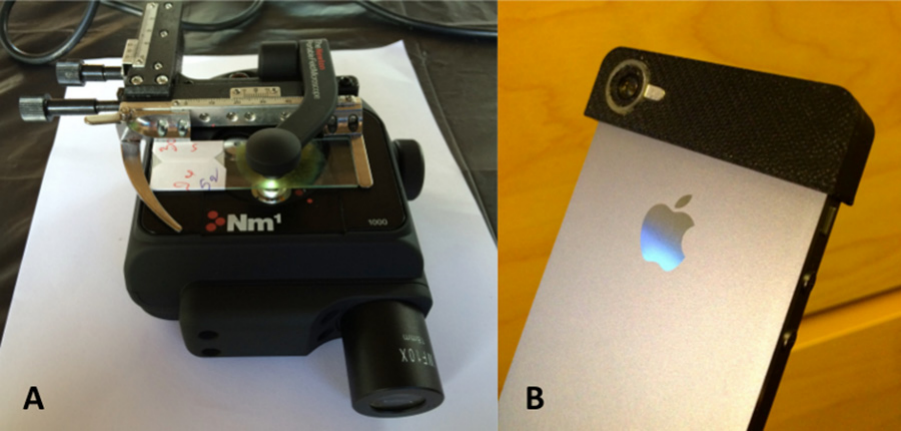
Handheld and mobile phone based microscopes. (A) The Newton Nm1-600 XY portable field microscope. (B) The reversed-lens CellScope attached to an iPhone 5s.

Slides were first evaluated by ‘gold’ standard microscopy, with an Olympus CX21 microscope under 10x and 40x lenses (Olympus; Volketswil, Switzerland). Laboratory technicians read slides, and 10% of all slides were re-examined by a senior expert microscopist (JTC, IIB) blinded to prior results for quality control and validation. Each slide was subsequently examined by two experimental microscopes; the Newton Nm1 Portable Field Microscope and the mobile phone-mounted reversed-lens CellScope (Fig 1). The Newton Nm1 microscope is a handheld, commercially available device, weighing 480 g with modular objective lenses (10x, 40x, and 100x), and has been described in field use elsewhere [11,13]. The reversed-lens CellScope fits a 3D printed plastic attachment weighing 5.2 g over an iPhone 5s (Apple; Cupertino, California, United States of America), with an embedded lens superimposed over the iPhone lens. This device harnesses the mobile phone’s light source to illuminate a specimen [14,16]. Laboratory technicians were provided with a half-day of training with direction on the operation of each microscope prior to initiating the study. This training consisted of didactic teaching sessions followed by supervised, hands-on training with multiple test slides. Briefly, the Newton Nm1 is operated by placing a slide in an XY translation stage mounted above the objective, focusing the objective on the sample, and then scanning the sample as it is viewed through the eyepiece of the microscope. The reversed-lens CellScope is operated by holding the mobile phone microscope above the sample and manually moving the device above the sample at the same time as maintaining focus and viewing the images on the screen. Since the slide evaluation was performed live using the screen, which displays images with a lower resolution than still photographs that capture the full resolution of the microscope, the effective resolution of the device used in this study was 14 μm.

### Microscopy Evaluation of Intestinal Protozoa Infection

One month after completion of the field study, SAF-fixed stool samples were subjected to an ether-concentration method, performed in the laboratory of the Centre Suisse de Recherches Scientifiques en Côte d’Ivoire near Abidjan, using a standard protocol [20]. In brief, the SAF-fixed stool samples were re-suspended and placed into a centrifuge tube and centrifuged for 1 min at 500 *g*. The supernatant was discarded and 7 ml of 0.85% NaCl plus 3 ml of ether were added to the remaining pellet. After shaking for 30 sec, the tube and its content were centrifuged for 5 min at 500 *g*. Finally, from the four layers formed, the three top layers were discarded. The bottom layer (including sediment) was placed on a microscope slide. One slide from each participant was created. Slides were examined by microscopy with an Olympus CX21 microscope (Olympus; Volketswil, Switzerland) by the same laboratory technicians for the presence or absence of intestinal protozoa, with 10% of the slides re-examined by an expert microscopist (JTC) for quality control. Identification of the presence or absence of intestinal protozoa was recorded. Laboratory technicians blinded to earlier results re-examined each slide with the Newton Nm1 microscope and recorded the presence or absence of intestinal protozoa. The clip-on version of the reversed-lens CellScope was not used for intestinal protozoa evaluation in the current study given its effective resolution of 14 μm, as described above.

### Statistical Analysis

Data were double entered into an Excel spreadsheet, transferred into EpiInfo version 3.2 (Centers for Disease Control and Prevention; Atlanta, Georgia, United States of America) and cross-checked. All analyses were conducted using R (R Foundation for Statistical Computing; Vienna, Austria). Prevalences were expressed as proportion and we calculated sensitivity, specificity, positive predictive value (PPV), and negative predictive value (NPV) of the experimental microscopes for each parasite, using conventional light microscopy as ‘gold’ standard. Using logistic regression models, we determined the sensitivity of the experimental microscopes for detecting any eggs, as a function of the egg count determined by conventional microscopy. Linear association of egg count estimates was assessed by Pearson’s correlation coefficient.

## Results

### Schistosomiasis and Soil-Transmitted Helminth Infection

One slide of filtered urine and a single Kato-Katz thick smear were examined from 226 individuals by conventional microscopy and the two experimental microscopes. Conventional microscopy identified 12 positive Kato-Katz thick smears for *S*. *mansoni* (5.3% prevalence), and 90 positive slides for *S*. *haematobium* (39.8% prevalence). No infections with *Ascaris lumbricoides* or *Trichuris trichiura* were noted. Hookworm eggs were detected in 22 of the Kato-Katz thick smears (9.7% prevalence). However, these were not further subjected to experimental microscopes given the concerns over rapid egg degradation after collection of stool samples and laboratory work-up, pending microscopy analysis, thus affecting the validity of our results [21].

**Fig 2:**
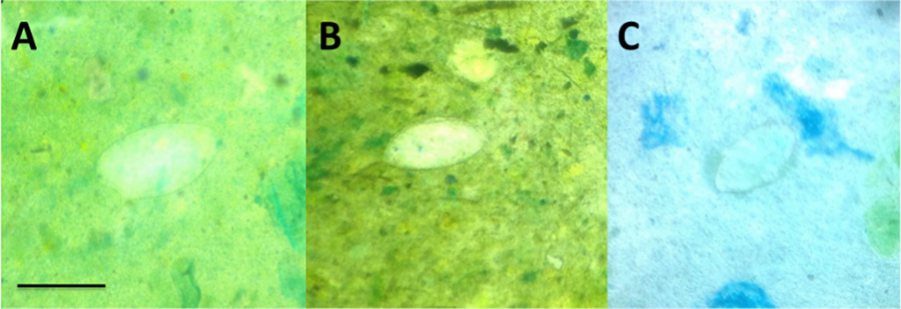
*S*. *mansoni* eggs visualized by different microscopes. (A) Conventional light microscopy with an Olympus CX21 microscope, (B) Newton Nm1 microscope, (C) reversed-lens CellScope. Bar = 100 μm.

**Fig 3:**
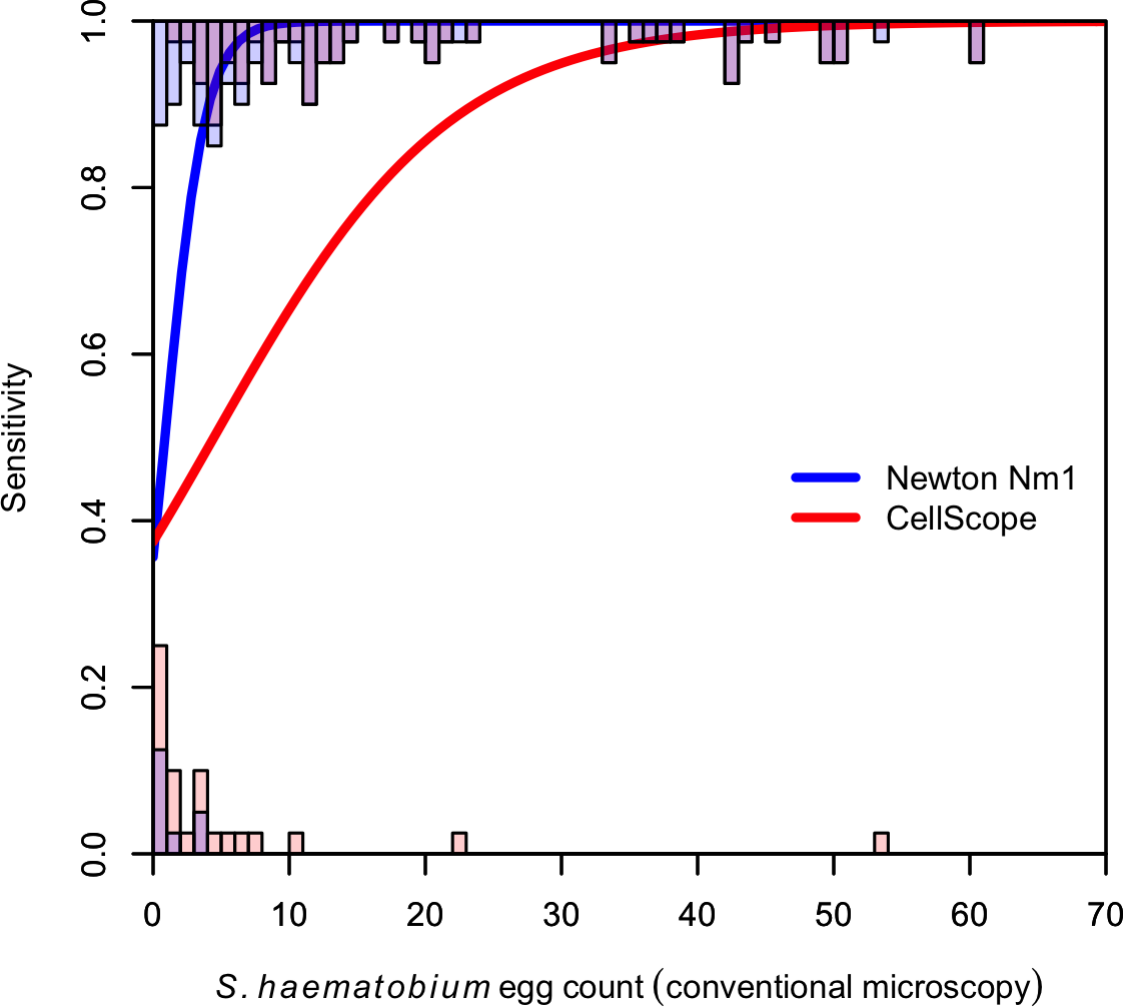
Sensitivity of handheld and mobile-phone based microscopes for *S*. *haematobium*. Modeled sensitivity of the Newton Nm1 portable microscope and reverse lens CellScope for diagnosis of *S*. *haematobium*, as a function of egg count determined by conventional light microscopy. Histograms indicate distribution of egg counts by conventional microscopy that were detected (top) and not detected (bottom), by Newton Nm1 (blue) and CellScope (red); purple shaded area indicates overlap.

Fig 2 demonstrates *S*. *mansoni* eggs visualized by conventional microscopy, the Newton Nm1 microscope, and a clip-on version of the reversed lens CellScope. Table 1 outlines the operating characteristics of the Newton Nm1 microscope and the reversed-lens CellScope for *S*. *mansoni* and *S*. *haematobium* diagnosis. Sensitivities for *S*. *mansoni* and *S*. *haematobium* with the Newton Nm1 microscope were 91.7% (95% confidence interval (CI) 59.8–99.6%) and 81.1% (95% CI 71.2–88.3%) respectively, and specificities were 99.5% (95% CI 97.0–100%) and 97.1% (95% CI 92.2–99.1%). *S*. *mansoni* and *S*. *haematobium* diagnosis with the reversed-lens CellScope demonstrated sensitivities of 50.0% (95% CI 25.4–74.6%) and 35.6% (95% CI 25.9–46.4%), respectively, and specificities of 99.5% (95% CI 97.0–100%) and 100% (95% CI 86.7–100%), respectively. The diagnostic sensitivity for the Newton Nm1 microscope for *S*. *haematobium* was 100% for egg counts ≥10 eggs/10 ml of urine. The clip-on version of the reversed-lens CellScope had limited sensitivity at low egg counts, but sensitivity improved as infection intensity increased, culminating in a sensitivity of >90% at 40 eggs/10 ml of urine or higher (Fig 3). Compared with conventional microscopy, estimates of egg counts for *S*. *haematobium* had a Pearson’s correlation coefficient of 0.98 using Newton Nm1 and 0.92 using the CellScope.

**Table 1:** Operating characteristics of handheld and mobile phone based microscopes for schistosomiasis. Operating characteristics of the Newton Nm1 portable handheld microscope and mobile phone reversed-lens CellScope integrated into routine use by laboratory technicians, compared with conventional light microscopy for the diagnosis of *S*. *mansoni* and *S*. *haematobium* (n = 226, Kato-Katz thick smears; n = 226 slides with filtered urine).

Organism	Conventional microscope, n (%)	Newton Nm1-600 XY portable field microscope				Reversed-lens CellScope			
		Sensitivity	Specificity	PPV	NPV	Sensitivity	Specificity	PPV	NPV
		(95% CI)	(95% CI)	(95% CI)	(95% CI)	(95% CI)	(95% CI)	(95% CI)	(95% CI)
*S*. *mansoni*	12 (5.3)	91.7	99.5	91.7	99.5	50.0	99.5	85.7	97.3
		(59.8–99.6)	(97.0–100)	(59.8–99.6)	(97.0–100)	(25.4–74.6)	(97.0–100)	(42.0–99.2)	(93.9–98.9)
*S*. *haematobium*	90 (39.8)	81.1	97.1	94.8	88.6	35.6	100	100	70.1
		(71.2–88.3)	(92.2–99.1)	(86.5–98.3)	(82.1–93.0)	(25.9–46.4)	(96.6–100)	(86.7–100)	(63.1–76.3)

CI, confidence interval; NPV, negative predictive value; PPV, positive predictive value

### Intestinal Protozoa Infection

Overall, 121 slides were examined for evidence of intestinal protozoa infection by both conventional microscopy and the Newton Nm1 microscope, with results outlined in Table 2. Based on conventional microscopy, the prevalence of *E*. *histolytica/E*. *dispar* and *G*. *intestinalis* of SAF-fixed stool samples subjected to an ether-concentration method were 4.9% and 20.7%, respectively. The Newton Nm1 microscope demonstrated a sensitivity for *E*. *histolytica/E*. *dispar* and *G*. *intestinalis* of 83.3% (95% CI 36.5–99.1%) and 84.0% (95% CI 63.1–94.7%), respectively, while specificity was 100%. For other intestinal protozoa, sensitivity of Newton Nm1 microscopy was variable (39–88%), while specificity was excellent (98–100%), as shown in Table 2.

**Table 2:** Operating characteristics of the Newton Nm1 portable handheld microscope for intestinal protozoa evaluation. Operating characteristics of the Newton Nm1 portable handheld microscope compared with conventional light microscopy for the diagnosis of intestinal protozoa infection (n = 121, Kato-Katz thick smears)

Organism	Conventional microscope, n (%)	Newton Nm1-600 XY portable field microscope			
		Sensitivity	Specificity	PPV	NPV
		(95% CI)	(95% CI)	(95% CI)	(95% CI)
*Entamoeba histolytica/E*.* dispar*	6 (4.9)	83.3	100	100	99.1
		(36.5–99.1)	(96.0–100)	(46.3–100)	(94.6–100)
*Giardia intestinalis*	25 (20.7)	84.0	100	100	96.0
		(63.1–94.7)	(95.2–100)	(80.8–100)	(89.5–98.7)
*Blastocystis hominis*	21 (17.4)	57.1	100	100	91.7
		(34.4–77.4)	(95.4–100)	(69.9–100)	(84.5–96.9)
*Chilomastix mesnili*	12 (9.9)	50.0	100	100	94.8
		(25.4–74.6)	(95.8–100)	(51.7–100)	(88.5–97.9)
*Endolimax nana*	23 (19.0)	39.1	98.0	81.8	87.3
		(20.5–61.2)	(92.1–99.6)	(47.8–96.8)	(79.2–92.6)
*Entamoeba coli*	56 (46.3)	87.5	100	100	90.1
		(75.3–94.4)	(92.9–100)	(90.9–100)	(80.2–95.6)
*Jodamoeba bütschlii*	10 (8.3)	80.0	100	100	98.2
		(44.2–96.5)	(95.8–100)	(59.8–100)	(93.1–99.7)

CI, confidence interval; NPV, negative predictive value; PPV, positive predictive value

## Discussion

Our study shows that handheld microscopes such as the Newton Nm1 portable field microscope and the mobile phone-based CellScope can be successfully implemented into public health settings, after minimal training of laboratory technicians, for the diagnosis of gastrointestinal parasitic infections in rural African settings. Novel diagnostic approaches for common parasitic infections could have a positive impact on the quality of care delivered in resource-constrained settings [3]. Handheld microscopes may be useful tools in such settings as they are lightweight and easily transportable, enabling the delivery of quality diagnostics to individuals in rural, remote, or under-serviced locations rather than transporting people or specimens to distant laboratories. In addition, these devices are battery powered and are helpful in settings where there is no or only intermittent electricity.

Indeed, mobile phone-based microscopes have several attributes that make them attractive for use in epidemiologic and public health settings. For example, mobile phone microscopes have the capacity to digitize images such that they can be saved and easily catalogued, or rapidly sent to other practitioners [22]. Digitization of images also allows for the attachment of geographic coordinates that may aid in mapping of infectious diseases and risk profiling of neglected tropical diseases [23]. Lastly, valuable clinical information associated with each image can be stored and catalogued, enabling healthcare providers for patient management. Additionally, the digitization of samples via mobile phone microscopy allows for computer vision and machine learning technology to aid in automated diagnoses and quantification of infectious diseases, such as malaria [24], schistosomiasis [22], giardiasis [25], and filariasis [26]. One potential barrier to widespread implementation is that handheld and mobile phone microscopy only addresses the issue of enabling microscopy in underserviced settings. Developing simple, reliable, and low-cost approaches to standardized sample and slide preparation are required as well, and have received comparably little attention.

To date, virtually all studies have evaluated handheld and mobile phone microscopes either in controlled laboratory settings or as used by expert microscopists. Prior to broader implementation, such devices must be rigorously validated in real-world settings and operated by front-line healthcare professionals. Our data confirm and add to findings from previous studies [9] demonstrating that laboratory technicians can reliably use handheld microscopes after minimal training. The Meade Readview handheld microscope was used by laboratory technicians in a Ugandan field study for *Schistosoma* diagnosis and demonstrated a sensitivity and specificity of 85% and 96%, respectively, compared to conventional microscopy. However, this device was limited by a smaller field of view, limited movement of the stage, and has not demonstrated widespread scale-up since its introduction [9]. Similarly, Ugandan laboratory technicians were trained to operate the Newton Nm1 microscope (as used in this study), to evaluate a set of pre-selected malaria slides, and demonstrated a sensitivity and specificity of 93.5% and 100%, respectively [11], although this study was not conducted in a true field setting. Our study adds to this prior work by implementing and evaluating the handheld microscope devices in a real-world field setting, demonstrating the utility of this device in day-to-day community-based diagnostic testing.

In the current study, the clip-on version of the reversed-lens CellScope demonstrated excellent diagnostic specificity for *S*. *mansoni* and *S*. *haematobium* infection, but only modest sensitivity for these trematode eggs. This observation is consistent with a prior study evaluating the reversed-lens CellScope for *S*. *haematobium* diagnosis [14]. We suspect sensitivities were low because users must manually hold the device and guide the lens over the entire surface area of a slide. The CellScopes used in this study were not anchored to a solid structure nor did they utilize a microscope stage such as with conventional microscopy or the Newton Nm1 microscope (Fig 1), however future iterations of this device will have the ability to reliably read an entire slide. Hence, the operators are likely undercounting schistosome eggs due to the challenges of manually maneuvering the device over a microscope slide. Interestingly, the CellScope rapidly gains sensitivity at higher egg counts. For example the sensitivity of CellScope reaches that of the Newton Nm1 microscope (>95%) for *S*. *haematobium* diagnosis when used by laboratory technicians at infection intensities of 40 eggs per 10 ml of urine, which is still considered to be a low-intensity infection (Fig 3) [27]. Diagnosing moderate- and high-intensity infections may be useful in clinical settings where worm burdens closely correlate with symptoms [28, 29]. However it is still crucial to have the most sensitive tests available to diagnose even very low intensity infections for disease mapping, epidemiologic surveys, particularly after drug interventions, and rigorous surveillance. Newer versions of the CellScope are currently in development that will enable automated sample scanning and image interpretation.

Limitations of our study include only commenting on the presence or absence of intestinal protozoa rather than quantifying these organisms. Future work should evaluate the diagnosis of intestinal protozoa in field settings with experimental microscopes rather than under laboratory conditions. Also, there were no infections with *A*. *lumbricoides* or *T*. *trichiura* in this setting, and hence, it would be useful to validate the diagnostic performance of these devices for these nematode eggs in other epidemiologic settings given the considerable global health importance of soil-transmitted helminthiasis [2], in addition to other endemic infections. Lastly, our study was restricted to a community-based setting, and future studies should validate the diagnostic capabilities of these devices in clinical environments.

In conclusion, handheld light microscopes have considerable potential for use in clinical and public health settings in resource-constrained environments. The clip-on reversed-lens CellScope, while convenient and low-cost to produce, was only modestly sensitive as currently used, however improvements are under development that could make it more appropriate for field deployment in the future. The Newton Nm1 handheld microscope, on the other hand, had good sensitivity and excellent specificity, and hence, could be readily integrated into real-world public health settings to diagnose intestinal parasitic infections.

## Supporting Information

S1 Checklist STARD checklist. (DOC)
